# Retrospective Analysis of 1168 Cases of Ovular Decidual Tissue from First-Trimester Abortions: Proposal for a Histopathological Diagnostic Framework

**DOI:** 10.3390/diagnostics16081128

**Published:** 2026-04-09

**Authors:** Eleonora Nardi, Vincenzo Arena

**Affiliations:** Area of Pathology, Department of Woman and Child Health and Public Health, Fondazione Policlinico Universitario A. Gemelli IRCCS, Istituto di Anatomia Patologica, 00168 Rome, Italy; eleonora.nardi@unicatt.it

**Keywords:** spontaneous pregnancy loss, abortion, histological examination, decidual–ovular material

## Abstract

**Background**: Early pregnancy loss, defined as the spontaneous loss of a pregnancy before 20 weeks of gestation or when the fetus weighs less than 500 g, remains a common obstetric complication, affecting up to 15% of clinically recognized pregnancies. Chromosomal abnormalities, particularly aneuploidies such as trisomies and monosomy X, account for 50–60% of first-trimester losses, with incidence increasing alongside maternal age. Additional risk factors include maternal medical conditions, uterine anomalies, infections, and modifiable lifestyle factors. Pregnancies conceived through assisted reproductive technologies also carry a slightly higher risk of miscarriage, often influenced by maternal age and embryo quality. **Methods:** Two pathologists, blinded to each other’s assessments, analyzed abortive material from patients who experienced spontaneous first-trimester abortion between January 2012 and January 2025 at Agostino Gemelli Hospital, Rome, Italy. Inclusion criteria were defined independently of patient demographics. No restrictions were applied regarding maternal age. With respect to gestational age, only first-trimester miscarriages (≤12 weeks of gestation) were considered. In cases of discordance, the case was reviewed and re-evaluated to reach a final diagnosis. **Results**: The findings of this study are presented as a proposed histopathological classification and diagnostic framework for first-trimester miscarriages. Specifically, a total of 1168 cases were categorized into eight distinct groups of miscarriage etiology based exclusively on the histomorphological features of chorionic villi and maternal decidua. **Conclusions**: Histopathological examination of products of conception is essential for confirming intrauterine pregnancy, identifying underlying maternal or fetal causes, and guiding future reproductive management, particularly in recurrent pregnancy loss. This study evaluates histopathological features of first-trimester losses, classifies findings by etiology, and proposes a practical diagnostic guide to support clinical decision-making and improve outcomes in subsequent pregnancies.

## 1. Introduction

The World Health Organization defines abortion as “the expulsion or extraction from its mother of a fetus or embryo weighing 500 g or less” [1].

Currently, abortion is defined as a spontaneous loss of a pregnancy [1] before the fetal viability, which is considered to be before 20 weeks of gestation or when the fetus weighs 500 g or more [2].

Worldwide, around 23 million pregnancy losses occur every year [3].

Spontaneous abortion is one of the most common first-trimester complications, affecting over 15% of pregnant women of reproductive age [4].

Approximately 50–70% of spontaneous conceptions are lost before the end of the first trimester; with most of them occurring early in pregnancy [5], primarily before 8–9 weeks of gestation [3].

Overall, almost 11% of women experience at least one pregnancy loss, and 1.9% and 0.7% have two or three pregnancy losses, respectively [3].

The risk of pregnancy loss decreases with increasing gestational age, with fetal loss after 11 weeks being less than 3% [5].

EPL can be theoretically divided into three categories based on etiology [4,6]:

• Inevitable loss due to an intrinsically abnormal gestation (e.g., abnormal karyotype);
• Failure to maintain an otherwise normal gestation due to underlying maternal physiologic or structural problems;
• Pathologic elimination of a normal gestation due to an active maternal disease process (e.g., massive perivillous fibrin deposition, chronic histiocytic intervillositis, chronic villitis and decidual vasculitis).

There are various possible causes of first-trimester miscarriage.

One of the most common risk factors for early pregnancy loss (EPL) is advanced maternal age.

For instance, the abortion rate for women aged 20–30 is 9% to 17%, but this rises to 80% at 45 years old [7,8].

Other contributing factors include chronic maternal disease (e.g., diabetes, hyperprolactinemia, and autoimmune conditions), infections (e.g., syphilis, Zika virus and cytomegalovirus), and structural uterine abnormalities.

Modifiable lifestyle-related risk factors—such as alcohol consumption, smoking, cocaine use and exposure to environmental toxins—are also linked EPL [7].

Nearly 50–60% of early pregnancy losses are associated with fetal chromosomal abnormalities. The earlier the loss occurs, the more likely it is due to chromosomal defect [5].

There is a heterogeneous distribution of karyotype anomalies among these disorders [9]. Aneuploidy, defined as the presence of an abnormal number of chromosomes, are the main causes occurring in an early embryogenesis [3,10]; particularly, trisomies are the most common chromosomal abnormalities (50% to 52%), followed by monosomy X (15% to 24%), triploidy (15% to 22%), structural rearrangements (4% to 8%), and tetraploidy (2% to 7%) [5,9].

Chromosomal translocation and inversions are sporadic (2%) in sporadic miscarriage but are a possible cause of recurrent losses [5].

Even though, in the majority of cases karyotype anomalies are de novo mutations and their impact on the risk in subsequent pregnancies is compound, the identification of the cause of abortion also plays an important role in genetic counseling [10].

The incidence of chromosomal abnormalities increases with maternal age, which contributes to age-related infertility [3].

Other causes of EPL include endocrine and maternal diseases, anatomical abnormalities of the female genital tract, infections, immune factors, hereditary disorders and trauma [5].

Assisted reproductive technology (ART) pregnancies (e.g., in vitro fertilization (IVF), Intracytoplasmic Sperm Injection (ICSI), egg donation) generally are characterized by a slightly higher risk of miscarriage compared to natural conceptions [11].

As with any pregnancy, the risk of spontaneous abortion (miscarriage) can occur in pregnancies conceived through ART, but there are several factors that may influence the risk, including the woman’s age, the quality of the embryos and whether the pregnancy is a singleton or multiple [11].

The miscarriage rate in ART pregnancies ranges from 15% to 25%. Whereas, in the general population, the risk of EPL is about 10–20%. Also in ART pregnancies, the maternal age is one of the most significant factors affecting the risk of miscarriage [12].

Women who undergo IVF are often older (>35 years) [13].

In fact, as women age, the quality of their eggs declines, which can lead to chromosomal abnormalities in the embryos, and this is one of the leading causes of miscarriage.

For women under 35, the miscarriage rate after IVF is typically around 15–20%.

For women over 40, the risk increases significantly, with some studies suggesting the rate could be 30–50% or higher, depending on factors like egg quality and the use of donor eggs [14].

In addition, embryos created through reproductive technology, especially from women of advanced maternal age, may have higher rates of aneuploidy leading to miscarriage. This is why genetic screening of embryos has become more common [15].

As in natural pregnancies, factors such as underlying maternal medical conditions (e.g., endometriosis and uterine abnormalities) and lifestyle factors (e.g., alcohol use, smoking, obesity) play important roles in determining the risk.

Fortunately, after a first pregnancy loss, many couples manage to have successful subsequent pregnancies; nevertheless, 2–4% of them will suffer recurrent losses, often without a recognized cause [16].

The histopathological examination of the abortive material is crucial for the diagnosis and for the subsequent management of a patient who experienced a first trimester miscarriage [17].

This procedure is useful in documenting an intrauterine or extrauterine pregnancy, identifying an important and unsuspected disease affecting the mother or the embryo and diagnosing conditions that may recur in future pregnancies.

In addition, the histology can explain the adverse fetal outcome and help the management of future pregnancies [4].

A correct histopathological diagnosis of the possible cause plays and important role in the management and follow-up.

In some cases, this examination may be of some value in determining the possible causes of recurrent pregnancy loss, or it may show an unexpected pathology [1].

In summary, histopathological examination of first-trimester pregnancy loss is a valuable and often underutilized diagnostic tool. It enhances the clinician’s ability to determine the etiology of the loss, supports decision-making in subsequent pregnancies, provides reassurance and information to patients, and fulfills important legal documentation requirements. A structured and consistent approach to the pathological evaluation of EPL is essential for improving reproductive outcomes and patient care.

The aim of this paper is (1) to evaluate the histopathological features of the products of abortion in the first trimester; (2) to illustrate the major pathological findings according to the possible causes of the miscarriage; and (3) to propose a useful guide to the routine diagnosis.

The correct identification of the etiology of the pregnancy loss can give important prognostic and diagnostic recommendations to support future pregnancies.

### 1.1. Development of the Placenta

The placenta plays a crucial role in nourishing the fetus, providing oxygen, removing waste products, and supporting the hormonal changes that maintain pregnancy; thus, its correct development is crucial.

The placenta undergoes several stages of development throughout the pregnancy.

Its development begins around 5 to 6 days after fertilization, when the blastocyst implants in the uterine lining. The trophoblast—outer layer of the blastocyst—invades the endometrium and forms the chorionic villi, which represent the functional units of the placenta.

During the first month, the trophoblast differentiates into two layers: the cytotrophoblast and the syncytiotrophoblast.

By week 4, a “primitive” placenta is formed.

Around weeks 6–7, maternal blood perfuses the placenta via maternal vessels, enabling nutrient exchange.

By weeks 10–12, the placenta is fully functional and continues growing into the second trimester, reaching about 450 g at term [18,19].

Umbilical cord development begins around the third week of embryogenesis with the formation of the connecting stalk, which links the embryo to the trophoblast. By week seven, the umbilical cord is fully developed, consisting of the connecting stalk, the vitelline duct, and the umbilical vessels, all encased in Wharton’s jelly and surrounded by the amniotic membrane [20].

#### First-Trimester Physiological Morphology of the Placenta

**Figure 1:**
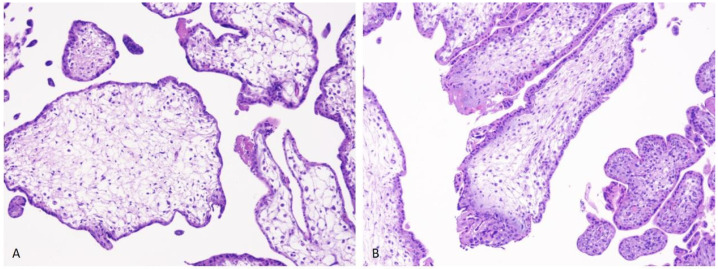
(**A**,**B**) During the first trimester, the chorionic villi are enlarged and enclosed by two cellular layers: the cytotrophoblast and the syncytiotrophoblast. At this stage, the blood vessels within the villi are not yet well developed. ((**A**,**B**) H&E staining; magnification 4×).

Histologically, the placenta in the first trimester (weeks 1–14) mainly consists of mesenchymal villi edematous and hypovascular—as well as a minimal number of immature intermediate villi [19] (Figure 1).

## 2. Materials and Methods

In this retrospective cohort study, we analyzed abortive material from patients who experienced spontaneous first-trimester abortion between January 2012 and January 2025 at Agostino Gemelli Hospital, Rome, Italy.

The material was analyzed at the Pathological Department of the same Institution.

After excluding 708 cases of recurrent abortion, a total of 1168 cases were included in the study.

Inclusion criteria were defined independently of patient demographics. No restrictions were applied regarding maternal age. With respect to gestational age, only first-trimester miscarriages (≤12 weeks of gestation) were considered. Case selection was based on histopathological adequacy: specimens were included if the sampled material had been entirely submitted for histological examination and was suitable for diagnostic evaluation. Specifically, eligibility required the presence of chorionic villi and maternal decidua, allowing a definitive histopathological diagnosis to be established.

The abortive material was received already fixed in 10% neutral buffered formalin. Upon arrival in the pathology laboratory, each specimen was macroscopically examined, appropriately sampled, and the weight of each sample was recorded. All available tissue was processed and embedded in paraffin to ensure complete histological evaluation. Paraffin blocks were then sectioned at approximately 5 µm thickness. The obtained sections were routinely stained with hematoxylin and eosin (H&E) for morphological assessment.

In selected cases, additional stains and immunohistochemical analyses were performed when clinically or morphologically indicated. In particular, in cases with suspicion of molar pregnancy, immunohistochemical staining for p57 was carried out to aid in the differential diagnosis. Similarly, in cases with histological features suggestive of cytomegalovirus infection, ancillary stains were performed as appropriate to support the diagnosis.

All cases were independently reviewed by two pathologists (VA and EN) and a joint review was performed for challenging cases. In cases of diagnostic uncertainty or discordance, the histopathological evaluation was re-assessed by the two pathologists (VA, EN) in a collegial manner. The re-evaluation process was initiated whenever one of the two observers had doubts regarding the initial histopathological interpretation. Each case was first independently reviewed in a blinded fashion with respect to the initial diagnosis. Subsequently, a joint review and discussion were performed to reach a consensus and establish the final diagnosis.

## 3. Results

The patients’ mean age was 35.8 years, and none had a prior history of recurrent pregnancy loss. The mean gestational age of the examined cases was 9 weeks and 3 days.

**Figure 2:**
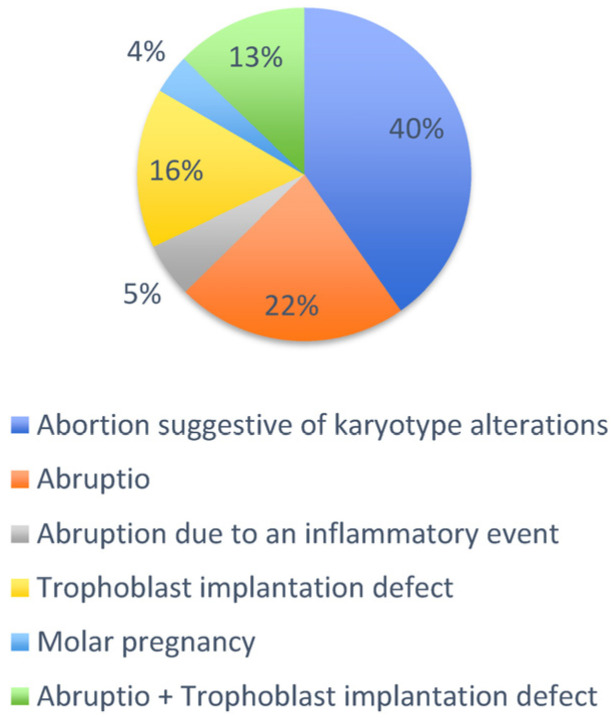
The graph illustrates the percentage distribution of suggested causes of early abortion among the 1168 cases included in the stud basing on the histological features.

Histological findings recorded in the database enabled the formulation of a diagnostic hypothesis, categorizing the possible causes of miscarriage as follows (Figure 2).

### 3.1. Histological Features in EPL Following Different Causes

#### 3.1.1. Abortion Due to Karyotype Alterations

Karyotype alterations can involve either the number or the structures of the chromosomes thus affecting normal fetal development and viability, often leading to spontaneous abortion.

Common numerical abnormalities include aneuploidy, trisomy, and monosomy; instead, structural chromosomal abnormalities include deletions, duplications, translocations, and inversions [21].

Women with a history of recurrent pregnancy loss may be more likely to have a chromosomal abnormality in their embryos. In these cases, chromosomal testing of the products of conception may help identify the underlying cause.

Among our cases, 40.2% showed histological features suggestive of karyotype alterations. In particular, in 27.8% of cases, the presence of a chromosomal abnormality was genetically confirmed (primarily triploidy). In the remaining cases, although no genetic confirmation was available, the histological characteristics did not allow allocation of the cases to any of the other categories.

##### Histology

Polymorphic, edematous and hypo/avascular chorionic villi with pseudo-cystic degeneration. Amniochorionic fragments, fetal red blood cells (RBCs), and necrotic decidua may be present.

All these findings are suggestive, but not conclusive, of karyotype alterations.

**Figure 3:**
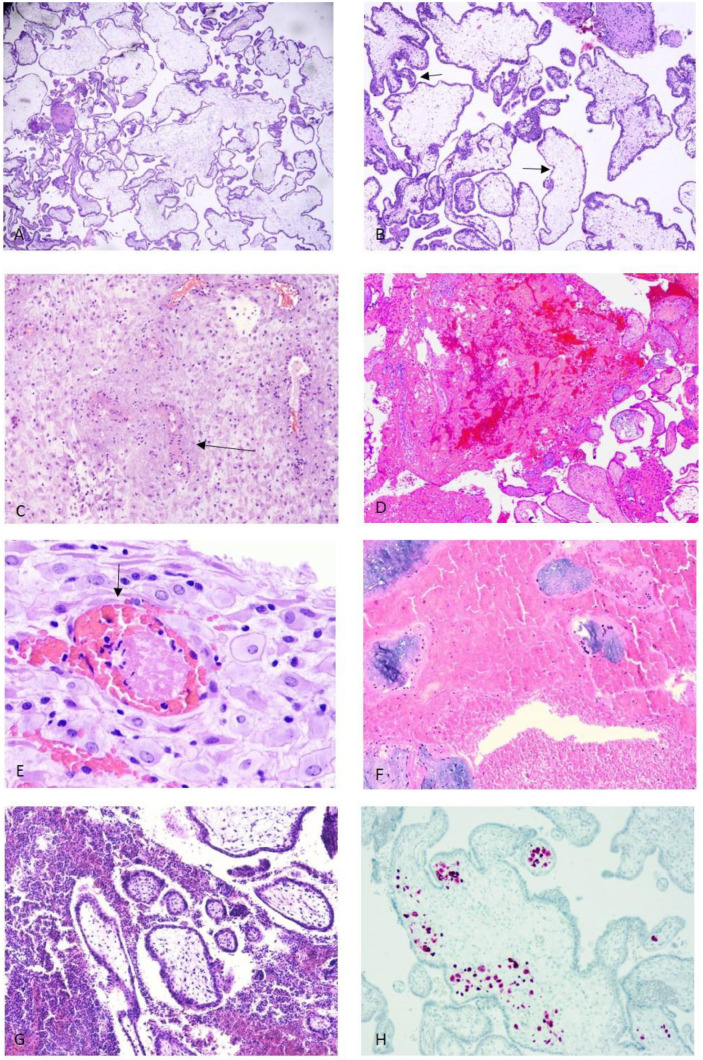
(**A**,**B**) Abortion due to karyotype alterations (9 + 5 weeks): dysmorphic villi with variable shapes and sizes (polymorphic and polymetric) and irregular outlines. Cytotrophoblast inclusions and pseudoinclusions are present (black arrow in (**B**)). (**C**) Trophoblast implantation defect (10 + 2 weeks): retention of muscular vessels layers (arrow) in the decidua. (**D**–**G**) Abruptio (11 + 3 weeks): ischemic villi and intervillous blood; thrombi in the decidua (black arrow in (**E**)); inflammatory infiltrates in (**G**). (**H**) Abortion due to Cytomegalovirus (CMV) infection: spontaneous abortion at 9 + 3 weeks. CMV-infected cells in villi (immunohistochemical staining). ((**A**–**H**): H&E staining; (**A**–**D**): magnification 4×; (**E**) magnification 10×; (**F**) magnification 4×; (**G**) magnification 4×; (**H**) magnification 10×).

A genetic test is then recommended (Figure 3A,B).

#### 3.1.2. Trophoblast Implantation Defect

Trophoblast implantation defects (TID) can cause an early pregnancy loss. These defects occur when the trophoblast cells fail to implant properly into the endometrium leading to insufficient placental development [22].

Several factors can cause a defective implantation including endometrial chronic inflammation or infections, autoimmune disorders (e.g., lupus or antiphospholipid syndrome), hormonal imbalances (particularly inadequate progesterone production), environmental factors (e.g., smoking, alcohol use, and obesity) and advanced maternal age.

##### Histology

Normal villar morphology with poor trophoblast invasion and retention of muscular vessels layers in the decidua suggests implantation failure (Figure 3C).

#### 3.1.3. Abruption

Abruptio placentae is a condition that can result in pregnancy loss, particularly in the later stages of gestation. It refers to a premature detachment of the placenta before delivery of the fetus caused by bleeding at the decidual placental interface [23]. Placental abruption occurs in 0.4% to 1.0% of all pregnancies [24]. This detachment deprives the fetus of oxygen and nutrients and potentially leads to pregnancy loss, preterm birth, and maternal hemorrhage [25]. Different risk factors can lead to placental abruption such as: abdomen trauma, preeclampsia, previous placental abruption, cocaine use, multiple pregnancy, advanced maternal age, and infection (e.g., chorioamnionitis) [25].

##### Histology

Ischemic villi, intervillous blood, fibrin, and thrombi are in the decidua.

Inflammatory infiltrates suggest a secondary inflammatory etiology (Figure 3D–G).

#### 3.1.4. Blighted Ovum

A blighted ovum, also known as anembryonic pregnancy, is a possible cause of early pregnancy loss and it is characterized by a gestational sac that forms and grows while an embryo fails to develop or stops developing early in the pregnancy [26]. The exact cause of a blighted ovum is still not clear, but it is believed to be due to chromosomal abnormalities or genetic errors that occur during fertilization. A transvaginal ultrasound is the most reliable way to diagnose a blighted ovum where no embryo is visible in the gestational sac.

##### Histology

Edematous chorionic villi with irregular contours and thinned trophoblast. The gestational chamber has a thinned amniotic lining in the absence of fetal red blood cells and embryonic residues.

#### 3.1.5. Exuberant Placental Site

Exuberant placental site is a rare condition in which abnormal placental tissue remains and grows in the uterus after a pregnancy has ended, leading to different complications and adverse outcomes [27]. This can occur after any pregnancy, but it is most associated with molar pregnancies. If an exuberant placental site is left untreated or if it evolves into gestational trophoblastic disease (GTD), it can lead to pregnancy loss or significant complications in subsequent pregnancies. 

##### Histology

**Figure 4:**
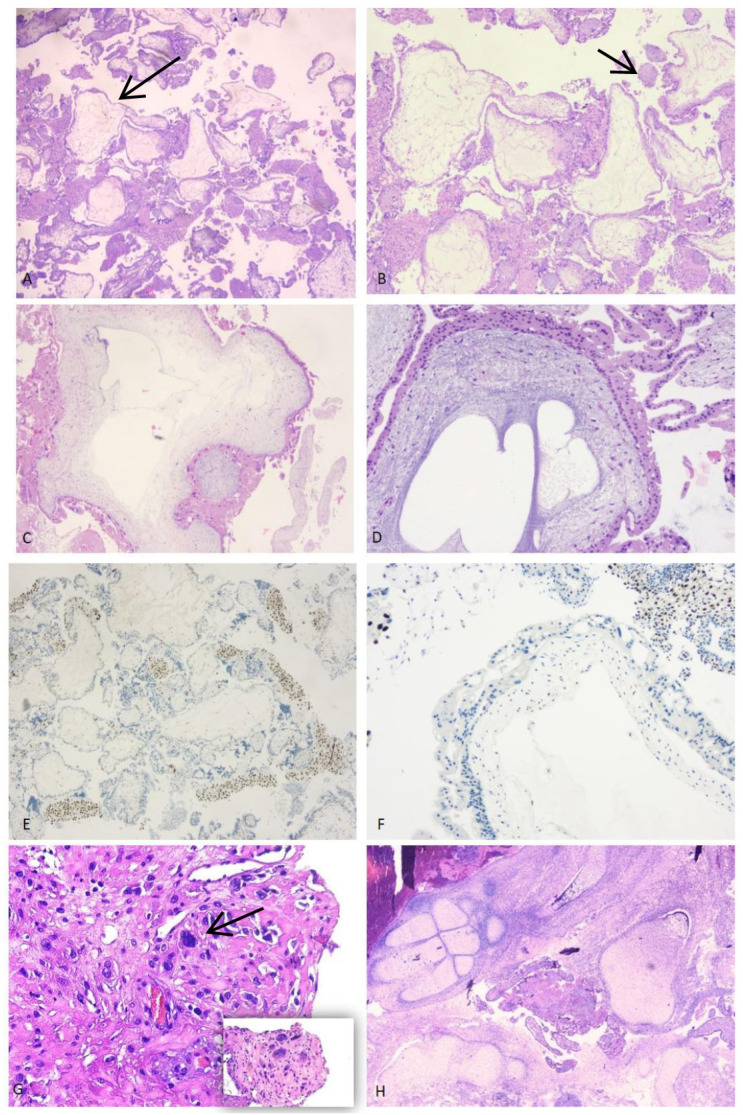
(**A**,**B**) Partial mole at 9 + 6 weeks: heterogeneity in villous size with two different populations (large and hydropic and small and fibrotic villi). Cistern formation can be seen (black arrow). (**C**,**D**) Complete mole at 10 + 3 weeks: hydropic villi, stromal cisterns and circumferential trophoblastic hyperplasia are identified. (**E**) Partial mole: p57 nuclear staining is present in the cytotrophoblasts and stromal cells. (**F**) Complete mole: p57 is negative in villous cytotrophoblast and stromal cells. The maternal decidua serves as internal positive control. (**G**) Exuberant placental site: mononucleated and multinucleated intermediate trophoblastic cells with eosinophilic cytoplasm and irregular nuclei (black arrow) invading myometrium. (**H**) Embryonic tissues in a spontaneous abortion at 13 + 2 weeks. ((**A**–**H**) H&E image; (**A**) magnification 4×; (**B**–**F**,**H**) magnification 10×; (**G**) magnification 20×).

Polymorphic and polymetric as well as edematous and hydropic chorionic villi. Decidua flaps show smooth muscle fibers, referable to the myometrium, mixed with elements of extravillous trophoblast (Figure 4G).

Though benign, follow-up is required.

#### 3.1.6. Partial Molar Pregnancy

A partial molar pregnancy (PMP) is a gestational trophoblastic disease that can cause EPL due to abnormal development of both the placenta and fetus. It is characterized by a triploid karyotype [28]. The placenta tissue grows abnormally, forming cysts, and cannot properly support the fetus. The lack of adequate placental support typically leads to miscarriage.

##### Histology

Polymorphic and polymetric chorionic villi with deep incisions in the trophoblastic lining and focal non-polar proliferation, pseudo-cystic degeneration, and presence of stromal cisterns. A second population of small and fibrotic villi is present. Fetal elements may be seen (Figure 4A,B).

Immunostaining for p57 (nuclear expression) is positive in at least 10% of villous stromal cells and cytotrophoblasts (Figure 4E).

#### 3.1.7. Complete Molar Pregnancy

A complete molar pregnancy (CMP) is a gestational trophoblastic disease that almost always leads to pregnancy loss during the first trimester. It occurs when there is an abnormal fertilization of an egg, resulting in an abnormal growth of trophoblastic tissue [29].

The abnormal proliferation of the trophoblastic cells leads to the formation of large fluid-filled cysts. Thus, the placenta cannot carry out its normal functions of nutrient and oxygen exchange, leading to inadequate placental support for any potential fetal development.

In addition, unlike a partial molar pregnancy, a complete molar pregnancy typically has no fetal development.

With proper treatment and follow-up, the prognosis is generally excellent, and women can go on to have future pregnancies [30].

##### Histology

Festooned villi, circumferential trophoblast proliferation, stromal cisterns, and nuclear atypia are identified. No embryo develops (Figure 4C,D).

Immunostaining for p57 (nuclear expression) is negative in cytotrophoblast and villous stroma (Figure 4F).

In some cases, histopathological evaluation of decidual–ovular tissue revealed the presence of embryonic components (Figure 4H).

In Table 1 are reported the most frequent causes of first-trimester miscarriage with their prevalent histopathological findings and reports.

## 4. Discussion

Early pregnancy loss (EPL) remains one of the most frequent and distressing complications in the first trimester of pregnancy, representing a major clinical concern for gynecologists and a significant emotional burden for patients [1]. While many early miscarriages—particularly those caused by chromosomal anomalies—are biologically unavoidable, identifying the underlying cause of EPL is essential for guiding future clinical management, patient counseling, and psychological support.

Histopathological examination plays a pivotal role in the diagnostic evaluation of EPL. It serves multiple purposes: confirming the presence of intrauterine pregnancy, distinguishing gestational from non-gestational tissue, and identifying critical maternal or embryonic pathologies. These include infections, molar pregnancies, chronic inflammatory conditions (e.g., chronic villitis or chronic histiocytic intervillositis), and placental abnormalities such as massive perivillous fibrin deposition or decidual vasculopathy, which may otherwise go undetected. Recognizing such abnormalities is particularly important in cases of recurrent pregnancy loss, where targeted interventions may be required. Moreover, histological assessment can uncover unsuspected maternal or gestational conditions that may not be clinically apparent but have prognostic significance for future pregnancies. For example, the detection of molar tissue necessitates follow-up for gestational trophoblastic disease, while identification of inflammation or vascular pathology may indicate underlying systemic disorders such as autoimmune disease or thrombophilia.

The value of histopathological analysis extends beyond diagnosis. It offers patients and providers concrete information regarding the nature of the loss, contributing to emotional closure and aiding in the grieving process. From a medico-legal standpoint, histological documentation provides objective evidence of intrauterine pregnancy and may help in the defense or clarification of medical decisions made during pregnancy management.

Despite its importance, histopathological examination is not uniformly performed following EPL. This variability underscores the need for standardized guidelines recommending routine submission of products of conception for histological evaluation, especially in cases where the clinical diagnosis is uncertain, the pregnancy was achieved via assisted reproductive technology, or the patient has a history of recurrent losses.

The main strength of our study lies in the relatively large sample size of first-trimester pregnancy loss cases, which enhances the robustness of the morphological observations. Additionally, the histopathological evaluation was performed by two pathologists, with discordant or uncertain cases undergoing blinded re-evaluation followed by joint discussion, thus increasing diagnostic consistency and reliability.

The principal limitation of the study is the lack of correlation with clinical characteristics of the patients. However, it is important to emphasize that the primary objective of this work was to develop and propose a structured framework to guide histopathological reporting in first-trimester pregnancy loss. For this reason, the study was intentionally focused on morphological standardization rather than clinicopathological integration.

## 5. Conclusions

Early pregnancy loss, while often unavoidable, warrants thorough evaluation to guide future clinical care and offer patients clarity and support. Histopathological examination of first-trimester pregnancy loss plays a critical role in identifying intrauterine gestation, uncovering underlying pathological processes, and informing management strategies—particularly in cases of recurrent loss or high-risk pregnancies.

By integrating histology into routine diagnostic protocols, clinicians can improve the accuracy of EPL assessments, offer more personalized care, and better counsel patients regarding prognosis and recurrence risks.

A structured and systematic histopathological approach should therefore be considered a standard component of EPL management, contributing meaningfully to diagnostic precision, patient outcomes, and reproductive planning.

## Figures and Tables

**Table 1:** Possible cause of miscarriage and proposed pathology reports.

Possible Cause of Miscarriage	Pathology Report	Comment
Trophoblast implantation defect	Normal villi, poor trophoblast invasion	Suggestive of implantation failure
Karyotype alterations	Edematous, avascular villi with incisions and pseudo-cystic degeneration of the trophoblast.	Suggestive, not conclusive- genetic testing needed
Blind ovum	Edematous villi, no fetal elements	Consistent with anembryonic pregnancy
Abruption	Ischemic villi, blood, fibrin, thrombi in decidua	Consistent with placenta abruption.
Inflammatory abruption	Intervillous blood, inflammation	Indicates secondary inflammatory cause
Exuberant placental site	Hydropic villi, myometrium fibers, extravillous trophoblast	Requires follow-up
Partial molar pregnancy	Stromal cisterns, focal non-polar proliferation, pseudo-cystic degeneration. p57+	Suggestive of PMP
Complete molar pregnancy	Cystic villi, atypical trophoblast. p57−	Diagnostic of CMP

## Data Availability

The original contributions presented in this study are included in the article. Further inquiries can be directed to the corresponding author.

## Abbreviations

EPL	Early Pregnancy Loss
ART	Assisted Reproductive Technology
IVF	In Vitro Fertilization
ICSI	Intracytoplasmic Sperm Injection
H&E	Hematoxylin and Eosin
RBCs	Red Blood Cells
TID	Trophoblast Implantation Defects
GTD	Gestational Trophoblastic Disease
PMP	Partial Molar Pregnancy
CMP	Complete Molar Pregnancy
CMV	Cytomegalovirus
